# Is EEG Suitable for Marketing Research? A Systematic Review

**DOI:** 10.3389/fnins.2020.594566

**Published:** 2020-12-21

**Authors:** Andrea Bazzani, Silvio Ravaioli, Leopoldo Trieste, Ugo Faraguna, Giuseppe Turchetti

**Affiliations:** ^1^Institute of Management, Scuola Superiore Sant'Anna, Pisa, Italy; ^2^Department of Economics, Columbia University, New York, NY, United States; ^3^Department of Translational Research and New Technologies in Medicine and Surgery, University of Pisa, Pisa, Italy; ^4^Department of Developmental Neuroscience, Istituto di Ricovero e Cura a Carattere Scientifico (IRCCS) Fondazione Stella Maris, Pisa, Italy

**Keywords:** consumer neuroscience, neuromarketing, EEG, eletroencephalography, consumer behavior, decision-making

## Abstract

**Background:** In the past decade, marketing studies have greatly benefited from the adoption of neuroscience techniques to explore conscious and unconscious drivers of consumer behavior. Electroencephalography (EEG) is one of the most frequently applied neuroscientific techniques for marketing studies, thanks to its low cost and high temporal resolution.

**Objective:** We present an overview of EEG applications in consumer neuroscience. The aim of this review is to facilitate future research and to highlight reliable approaches for deriving research and managerial implications.

**Method:** We conducted a systematic review by querying five databases for the titles of articles published up to June 2020 with the terms [EEG] AND [neuromarketing] OR [consumer neuroscience].

**Results:** We screened 264 abstracts and analyzed 113 articles, classified based on research topics (e.g., product characteristics, pricing, advertising attention and memorization, rational, and emotional messages) and characteristics of the experimental design (tasks, stimuli, participants, additional techniques).

**Conclusions:** This review highlights the main applications of EEG to consumer neuroscience research and suggests several ways EEG technique can complement traditional experimental paradigms. Further research areas, including consumer profiling and social consumer neuroscience, have not been sufficiently explored yet and would benefit from EEG techniques to address unanswered questions.

## Introduction

In recent years, the idea of applying neuroscience techniques to marketing has given rise to a new field of research known as consumer neuroscience or neuromarketing, and to a multitude of specialized consultant companies. Their list of services includes predicting sales, discovering unconscious drivers of consumer behavior, and identifying preferences and willingness to pay. Eminent authors tried to systematize the proliferating works from this emerging field (e.g., Fortunato et al., 2014), but their first effort was entirely aimed to offer an overview of the variety of available tools and to eventually compare them, stressing limitations and plausible scenarios of application (Harris et al., 2018). Functional Magnetic Resonance Imaging (fMRI) and Electroencephalography (EEG) are the most frequently adopted neuroscientific techniques to address marketing questions. One of the main limitations in the analysis of studies on consumer neuroscience relied on a jargon barrier. Neuroeconomics, neuromarketing and consumer neuroscience are terms often confused in the common speech, and even among scholars of adjacent fields (e.g., Hubert and Kenning, 2008). Their boundaries remain undefined, since the field is so recent. The term neuromarketing was first introduced in 2002, followed by other definitions (see Fortunato et al., 2014 for a brief description of their history and diffusion). In 2008 Hubert and Kenning proposed to refer to consumer neuroscience as an academic literature definition, while maintaining neuromarketing for industry. Ramsøy et al. (2018) tried to resolve this definition ambiguity in order to clarify the underlying conceptual framework.

The aim of this systematic review is to respond to the need of focusing exclusively on studies applying EEG. It is beyond the purpose of this review to explain the methodological features and results obtained with other techniques, e.g., fMRI [discussed extensively in Ariely and Berns (2010) and Fortunato et al. (2014)]. A decade ago, Ariely and Berns (2010) separated “hope from hype” in the use of brain imaging applied to marketing and scaled down the legitimate expectations for companies that are willing to test innovative methods. The authors mainly referred to functional Magnetic Resonance (fMRI), but similar concerns might be raised with respect to the other neuroscientific techniques as well.

The need of focusing on EEG studies in marketing comes from the increasing number of works on consumer neuroscience using EEG published over the last few years, and it is reinforced by three specific reasons related to certain intrinsic characteristics of this technique. First, EEG data are characterized by high temporal resolution (milliseconds) compared to other brain imaging techniques (e.g., seconds for fMRI) (see Michel and He, 2010). This feature is commonly used by neuroscientists to classify techniques and it is critical for marketing research, as it permits the identification, within a functional time window, of neurophysiological correlates of the exposure to stimuli, such as advertisements (e.g., music and videos). Second, standard EEG devices are non-invasive and allow participants to act normally either in the lab (watching pictures or videos on a screen) or in a store (in the case of field experiments). Finally, the cost of EEG is significantly lower if compared to other brain imaging techniques, and the equipment required to conduct studies is commonly found in neuroscience departments. For these reasons, we consider EEG to be one of the most readable and promising neuroscientific tools for consumer neuroscience studies.

The final aim of our systematic review is to address researchers and managers who wish to explore the consumer neuroscience literature and are possibly considering starting using EEG for their marketing purposes, or who are interested in extending the use of EEG to other disciplines.

**Figure 1:**
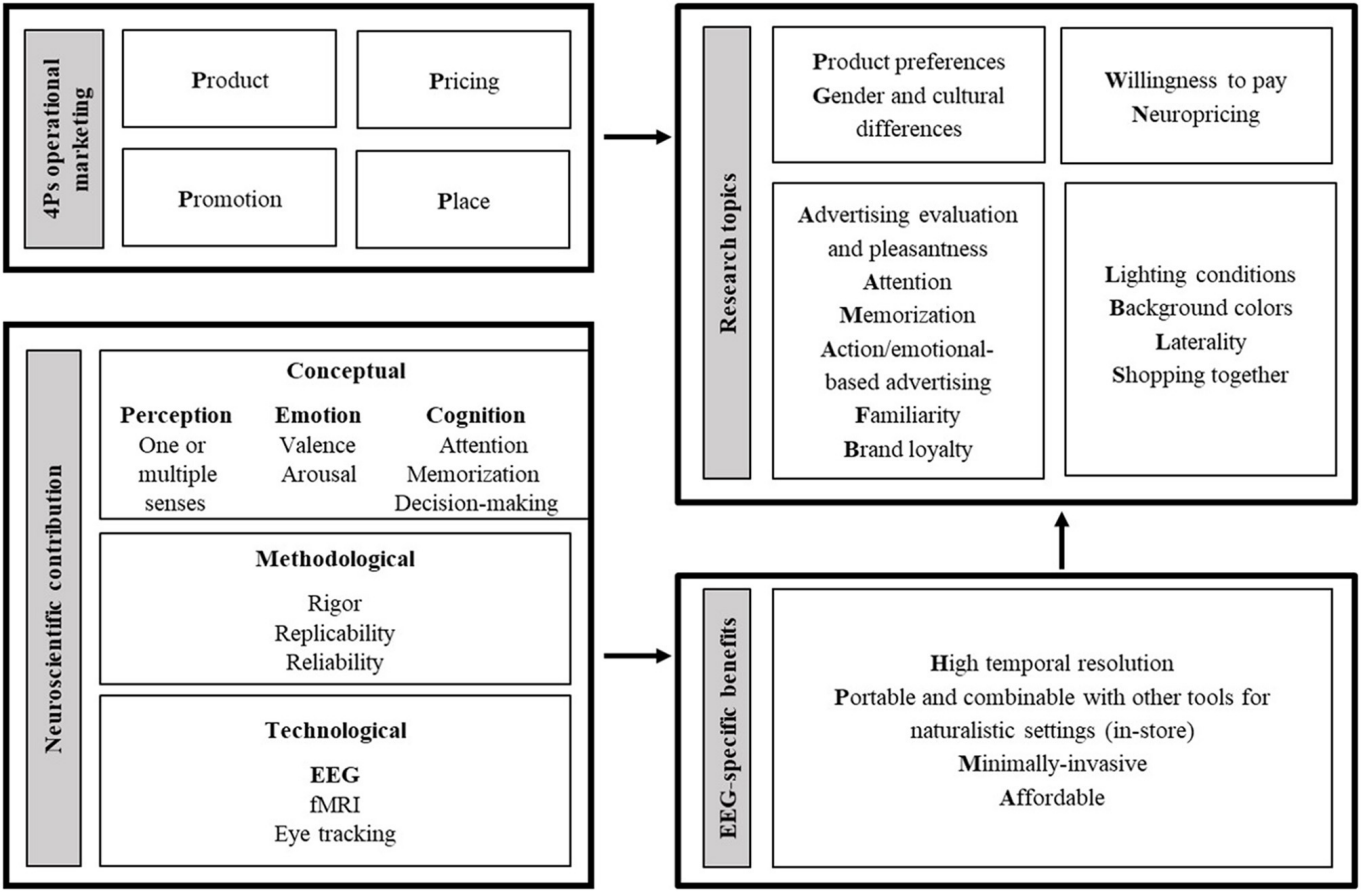
Conceptual framework. The four Ps of operational marketing indicate a map of the research topics ranging from brand loyalty to pricing. Research in this area can be improved by the adoption of tools from neuroscience. In addition to the technological contribution (and the EEG-specific strengths highlighted in this review), marketing researchers can also benefit from the conceptual and methodological contributions offered by the neuroscience literature.

We provide a conceptual framework of the contribution of neuroscience in terms of theoretical knowledge, methodological approach and technological implementation to marketing (see Figure 1). By assessing the mental states of the consumers, consumer neuroscience explains the cognitive, emotional, and perceptual structures underpinning human decision-making, thus conferring a higher degree of methodological rigor to the most common marketing research questions. Neuroscientific techniques offer a valuable support at every stage of the creative workflow process, from the creation of a concept, passing throughout the production of a product, its dissemination, to a service or a commercial; and yet, they are useful to verify the efficacy of an advertising campaign (Plassmann et al., 2012; Cerf and Garcia-Garcia, 2017; Opris et al., 2020). In the quest for brain-region-specific localization of mental functions, a versatile technique as EEG can become a compass for consumer thoughts. However, despite the proliferation of peer-reviewed articles in a similar variety of directions, even the most promising results suffer from limits in the interpretation and in the universal applicability of the evidence. In parallel, most neuroimaging approaches are affected by a reverse inference problem in the interpretation of the results. For a complete discussion of this aspect we direct the reader to consult papers that focus on the methodological challenges of neuroimaging (e.g., Poldrack, 2011; Hutzler, 2014).

The rest of the paper is structured as follows: a brief paragraph on EEG provides more details on the technique for those readers who are less familiar with this approach. The Method section contains a description of the search criteria (**Figure 3**), classifications (Table 1), and statistics regarding recent consumer neuroscience experiments (**Figures 4**, **5**). Next, the Results section offers a more detailed description of the main findings and managerial implications (**Table 3**). Finally, the Discussion section summarizes the main contributions, lists general indications about how to design an EEG experiment of consumer neuroscience, and offers our recommendations regarding future research (**Table 4**).

**Table 1:** Classification: topics and research questions.

**4 Ps**	**Research Topic**	**Keywords**
**PRODUCT**	*Product* characteristics and preferences	Liking, preference prediction, consumer profiling, and success prediction
	Gender and cultural *differences*	Differences (in preference, attention, or willingness to pay) across populations
**PRICE**	*Pricing* and willingness to pay	Estimation of price elasticity and willingness to pay
**PROMOTION**	*Advertising* rating and pleasantness	Evaluation of ads, comparison between EEG and traditional rating techniques
	Advertising attention and *memorization*	Cognitive processes (attention, memorization, evaluation) related to ads
	Rational and emotional *message*	Effectiveness of different types of messages
	*Brand* identity	Brand strategy, brand extension, and familiarity with the target product
**PLACE**	Product *context*	Packaging, lighting, and interaction with other consumers
**MISCELLANEOUS**		Residual category

*The words in italics are used in the following tables to refer to the research topics*.

### The Technique: Electroencephalography (EEG)

**Figure 2:**
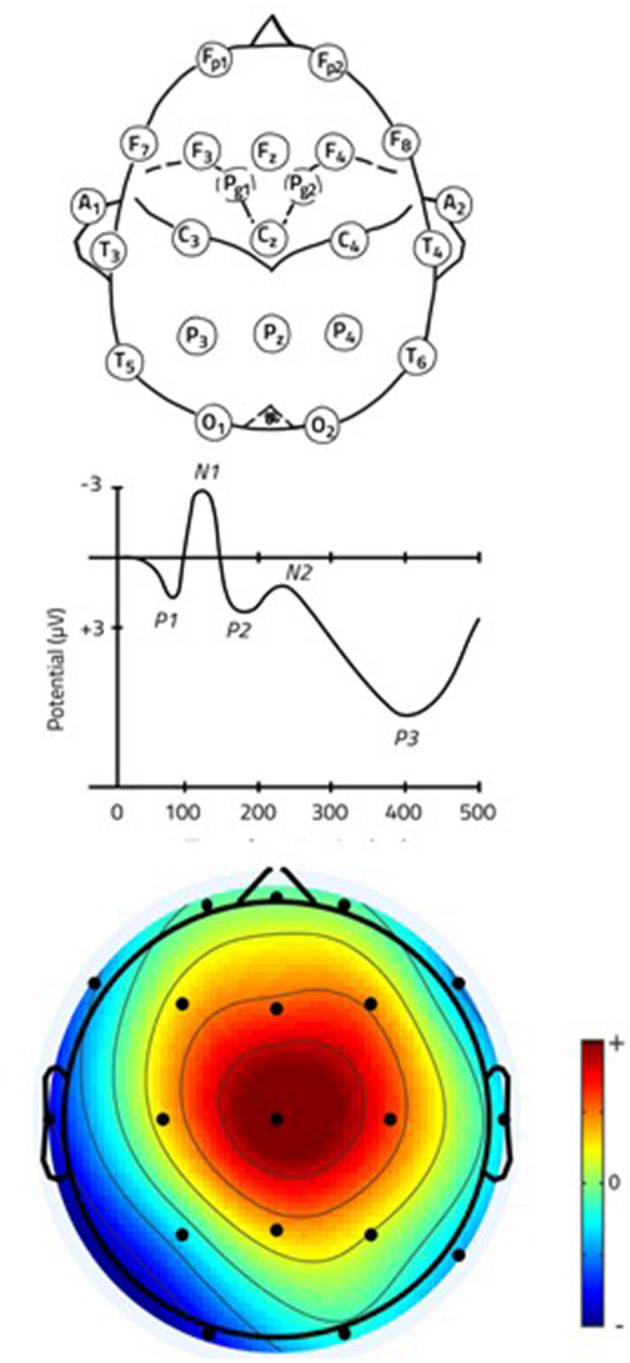
EEG set-up, ERPs and spectral map. Top: Position of 24 electrodes according to the International 10–20 system. Front to back: Fronto-polar, Frontal, Pharyngeal, Auricular, Central, Temporal, Parietal, Occipital (Sazgar and Young, 2019); Center: Event-Related Potentials (ERPs) classified based on latency with respect to the stimulus; Bottom: Spectral map showing EEG activation at P200 latency (the color code represents the topographic distribution of the voltage values).

EEG is a non-invasive electrophysiological technique based on brain-generated electrical waves measured via sensors (electrodes) applied to the scalp. Electrodes can be directly applied to the scalp or a head cap can be worn instead. Electrodes are located according to the international 10–20 system (see Figure 2) (Sharbrough et al., 1991). The number of sensors used for exploring brain activity can vary from one single electrode to hundreds. Electrodes measure the voltage of electrical potentials and the frequency of oscillations (Hz) in the brain activity. EEG is conventionally divided into fundamental frequency bands: delta (0.5–4 Hz), theta (5–7 Hz), alpha (8–14 Hz), beta (15–30 Hz), gamma (30–50 Hz), each typical of a rather specific behavioral state (Ahlert et al., 2006; Sanei and Chambers, 2007; Banich and Compton, 2011).

From its first recordings in the 1920s on human beings by Hans Berger (Berger, 1929), EEG received great attention in neuroscience for its versatility. For decades it represented the preferred approach to brain imaging, with a wide set of applications in neurology, neurosurgery, and psychiatry. Its high temporal resolution (milliseconds) called the attention of psychologists and social science scholars, who tried to apply EEG to study human decision-making (Heekeren et al., 2008). The analysis of Evoked Related Potentials (ERPs), which are early potentials with defined latencies (from <10 ms to more than 500 ms) (see Figure 2), opened interesting scenarios for the understanding of consumer decision-making. For an overview on the correspondence between neural correlates and marketing research topics explored we refer the readers to Harris et al. (2018). Electroencephalography data are rather complex to interpret, particularly when compared to behavioral data, as they include spatial and temporal dimensions of low amplitude electrical signals. Internal (e.g., eye-movements) and external (e.g., environmental noises) factors can influence EEG signals, that are hence commonly cleaned from artefacts by using dedicated computer software for amplification, filtering, preprocessing of raw data, and component analysis (see e.g., Urigüen and Garcia-Zapirain (2015) for an extensive review on artifact removal and Michel and Brunet (2019) for a practical review of the analysis steps). Each signal can, and often must, be separated into multiple components. Most of the studies include a power spectrum analysis. The signal from each channel (electrode) is broken down into multiple frequency bands. Alpha, beta and theta frequency bands are usually included in the analysis, and full analysis contains gamma and delta bands as well. The intensity of the signal associated with each channel and frequency can be compared across time. Alternatively, they can be used to create indexes. Given the large variability between research groups in the implementation of the aforementioned steps applied to EEG signal analysis, recent standardization efforts led to the publication of recommendations and guidelines for EEG and ERPs data processing (e.g., see Keil et al., 2014; Babiloniet al., 2020; Pernet et al., 2020). Previous EEG studies tried to associate EEG features to behavioral patterns, or even predictions, despite the potential problem in the interpretation of the results of the reverse inference (Poldrack, 2011; Hutzler, 2014). Such indexes are, for instance, vigilance (alpha band, Hoefer et al., 2016), emotional (Pozharliev et al., 2015), approach and avoidance motivation (alpha asymmetry, Kelley et al., 2017), arousal (beta band, Gupta et al., 2016), pleasantness (alpha and beta bands, Guixeres et al., 2017), and activation (alpha and beta bands, Bertin et al., 2013). The EEG metrics applied in consumer neuroscience, their predicted values, and their limitations have been comprehensively reviewed also by Hakim and Levy (2019). Since the location of the source remains the major limitation of EEG, estimation of cortical activity and reconstruction of 3D configurations are useful for improving the spatial accuracy of the analysis and identifying specific brain areas involved in a task. Useful techniques include the LORETA method (low resolution brain electromagnetic tomography, e.g., Cook et al., 2011) and Global Field Power (GFP, e.g.„ Kong et al., 2013).

The temporal dimension is also explored in different ways. Time frequency analysis focuses on the change over time of a signal, and our list of articles includes two different techniques: Event-Related Potentials (ERPs) and Steady-State Probe Topography (SSPT). ERPs extract the variation in the signal that is recorded at the beginning of each trial, aligned with a specific event or administration of stimulus (see Camarrone and Van Hulle, 2019). The intensity of the peak within a certain time window (for example 300 ms using a P300 analysis scheme) is compared under different conditions (see Uva et al., 2015). Since ERPs have amplitudes of the order of a few microVolts, occurring on background oscillations ranging up to 200 microVolts, the analysis needs to average multiple trials from each individual (see Srinivasan, 2007). This aspect can represent a limit for the experimental design. Steady-State Probe Topography (SSPT) has a high signal-to-noise ratio and overcomes this critical aspect, so data can be analyzed based on a single trial per individual (see Rossiter et al., 2001). Final results are extracted from frequency bands, indexes, cortical activities, and signal peaks by using statistical models that analyze differences between experimental conditions. Finally, decoding analysis has taken advantage of machine-learning algorithms to forecast behavior from brain imaging data; both spectral analysis and ERPs can be used as classifiers (see Fudali-Czyż et al., 2016).

For all these reasons, choosing the proper analysis technique is a craft that still requires experience and is strongly dependent on the type of information the experimenters want to extract from available data. Recent attempts of standardizing EEG data analysis have been made and are promising in the perspective of increasing the replicability of results (e.g., see Keil et al., 2014; Babiloniet al., 2020; Pernet et al., 2020).

## Method

### Systematic Review Protocol

We used a PICO framework to guide the literature search and determine the boundaries of the review (Schardt et al., 2007).

- Population: healthy adolescents or adult humans (potential consumers or users).
- Intervention: presentation and/or interaction with stimuli (audio, visual, audiovisual, tactile, olfactory, taste, or real product) while recording brain activity with an Electroencephalography (EEG) device.
- Comparators: Assessment: neutral task or presentation of neutral stimuli; Methodology: traditional measures (e.g., questionnaires) or combination of techniques (e.g., eye-tracking).
- Outcomes: assessment of brain activity patterns recorded using EEG related to consumer behavior.

**Figure 3:**
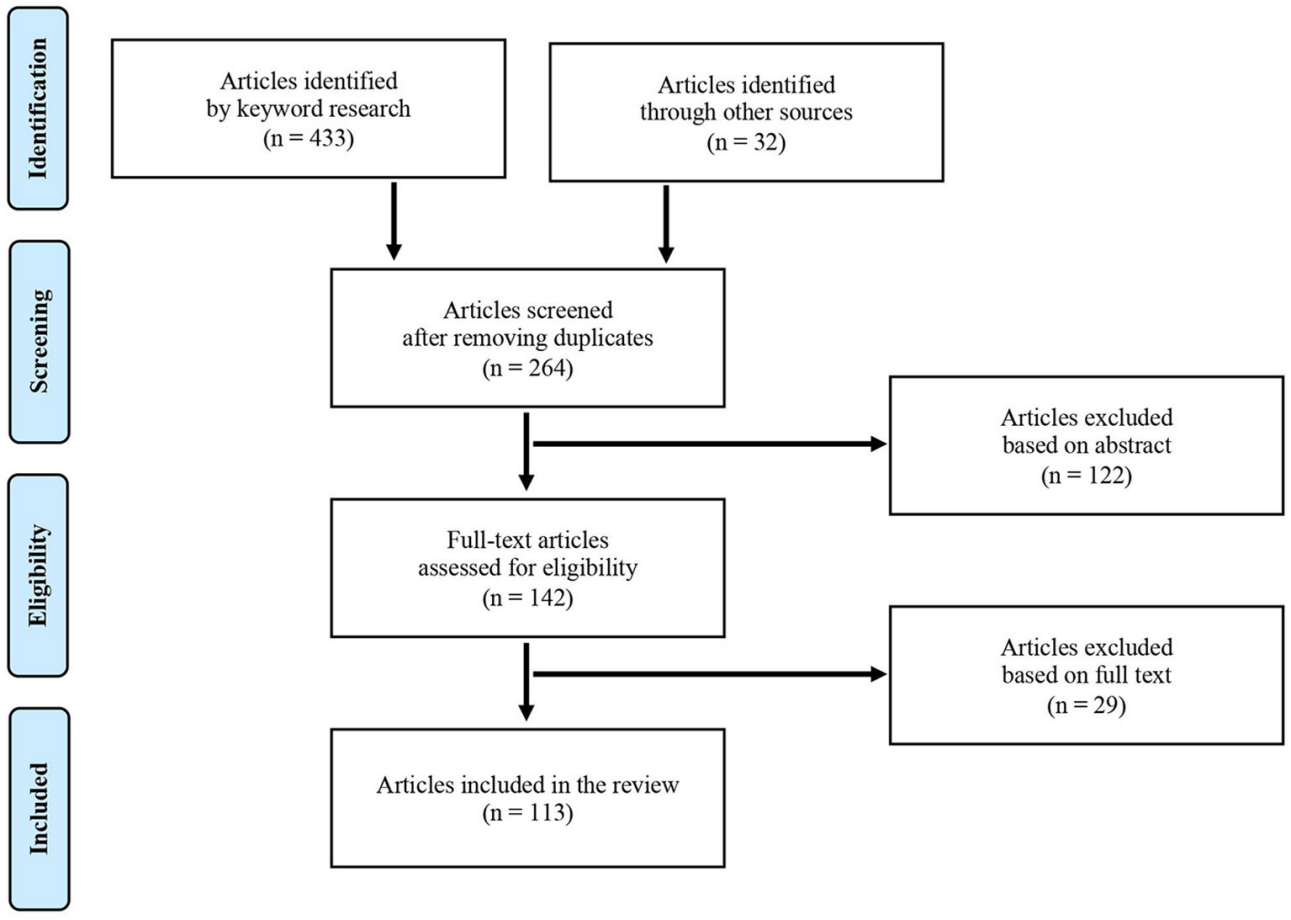
PRISMA flow diagram. The diagram indicates the number of articles in each step of the systematic review, from the initial identification to the application of the exclusion criteria.

We conducted a systematic review by consulting five datasets (Scopus, ISI Web of Science, PubMed, Emerald, and EconLit) and searching the terms and keywords EEG AND (Neuromarketing OR “Consumer neuroscience”). Based on preliminary research, the use of the term “EEG” instead of “electroencephalography” in the research leads to more numerous lists of results with few or no studies removed. We limited our research to the keywords “Neuromarketing” and “Consumer neuroscience” because it is beyond the scope of this review to include papers whose findings contributed to the development of the field without having a clear marketing focus. We screened 264 articles published between January 2000 and June 2020 and analyzed 113 of them selected based on the focus on marketing and consumer behavior research questions (see PRISMA diagram, Figure 3, based on Moher et al., 2009), summarizing the main results and coding the characteristics of the study (see Online Appendix).

The inclusion criteria led to the selection of original and peer-reviewed articles containing the description of the experimental design and the analysis of the data collected, with a marketing focus and based on collection of EEG data. The exclusion criteria ruled out studies not published in the English language, book chapters, conference proceedings, reviews, commentaries, and preliminary versions of studies by the same authors which were subsequently published as full articles. We also excluded papers that contained only a vague description of the experimental paradigm, insufficient for the replication of the study. We included 32 additional articles identified from the bibliographies of the papers consulted for this review, using the same inclusion criteria described above.

Finally, we want to clarify the reason we restricted our attention to articles published from 2000 onwards. Earlier articles (e.g., Rothschild and Hyun, 1990) are not easily comparable with current methodology because of their strong exploratory nature, the limited sample size, and the different methods used to display stimuli.

We assessed the quality of the articles selected grounded on: (a) the quality of the study design; (b) the number of citations; (c) the ranking of the journal where the paper was published. With respect to the study design, we assessed the studies using an adapted version of the “Quality Assessment Tool for Before and After Studies” (adapted from the National Health Institute NHLBI study quality assessment tools, see details in the Online Appendix). This method is based on the evaluation of several dimensions of the experimental study, including clarity of the research question and selection criteria, adequacy of the sample size, and completeness of the analysis. In the Online Appendix we reported, for each paper, the individual items and the final quality, the number of citations normalized by publication date from Google Scholar (updated in September 2020), and the ranking of the journal according to SCImago Journal Rank (SJR, September 2020).

### Classification Criteria

We classified the papers based on the research topic and the characteristics of the experimental paradigm, including study design and the type of stimuli used. Table 1 contains the classification of the articles by research topics. We started from the famous four Ps of marketing (Product, Price, Promotion, Place; McCarthy, xbib1960) and we divided each of them in sub-topics, indicating some common keywords.

In a similar manner, we considered the main characteristics of the experimental paradigm. Table 2 contains a variety of examples with the associated references.

**Table 2:** Experimental characteristics: tasks, stimuli, participants, and additional techniques.

**Experimental characteristic**	**Example**	**Description**	**Reference**
Tasks	Observation	The participant observes the stimulus and is not required to complete any specific task	Rossiter et al., 2001
	Questionnaire	The participant evaluates the stimulus based on one or multiple dimensions (e.g., Likert scales)	Rosenlacher et al., 2018
	Choices	The participant chooses one product or bundle of product out of a choice set	Ravaja et al., 2016
	Willingness to pay	The participant observes a product (e.g., image, price, and description) and indicates if the product is cheap or expensive	Lee, 2016
Stimuli	Audiovisual	TV commercials, movie trailers, movie clips, TV series scenes	Gupta et al., 2016
	Visual	Images, description, and price of a product, brands or logos, ads pictures, app screenshots	Ramsøy et al., 2018
	Real products	Interaction with the real product (e.g., brew coffee using a coffee machine)	Sargent et al., 2020
Participants	Balanced sample	Subgroups of participants with different age, gender, and/or income	Vecchiato et al., 2014b
	Target age/gender	Target group (e.g., young females) for the product category used as stimulus in the study	Baldo et al., 2015
	Segmentation	Participants recruited in two or more groups (e.g., based on the familiarity with the product)	Royo et al., 2018
Additional techniques	Eye tracking (ET)	Eye movements are used to investigate attention and acquisition of information	García-Madariaga et al., 2019
	Electromyography (EMG)	EMG is used to collect movements for facial muscles and infer expressions and emotions	Boshoff, 2016
	Galvanic Skin Response (GSR)	GSR indicates the electrodermal activity, whose changes can be caused by emotional stress	García-Madariaga et al., 2020

#### Tasks

The most common protocol involves displaying a list of stimuli (pictures, videos, or TV ads), but an increasing number of studies contains more sophisticated tasks, such as like/no-like ratings, evaluation with Likert scales, choices, or elicitation of the participants' willingness to pay. Measures of memorization and ease of recall are also possible by arranging multiple sessions.

#### Stimuli

Most of the studies adopt visual stimuli (e.g., product images, brands or logos, 42% of the reviewed articles), audiovisual (44%, e.g., TV commercials or movie trailers), or both (7%). More rarely we observe real products or other types of stimuli (7%, including tactile, olfactory, and taste stimuli).

#### Participants

The combination of research questions and type of stimuli often suggest what kind of population is chosen as the primary target for the study. Articles that provide detailed information about recruitment and categorization of the participants range from balanced samples (based on age, gender and/or income) to specific target groups selected based on demographic characteristics. Other studies focus on habitual consumers for a specific product category or analyze the differences between habitual and novel consumers. The number of participants for a study must be chosen carefully based on initial hypotheses, number of trials, expected magnitude of the effect from the literature, and power analysis. Across the studies in this review, we observe an average pool size of 34 participants, with a long tail (up to 331 participants), and larger pools usually observed for studies published in high-rank journals (43 for the top SJR quartile, 30 for the bottom quartile).

#### Additional Techniques

EEG is compatible with a wide range of tools to collect non-behavioral data, and 32% of the experiments combine EEG with eye tracking or electrooculography (ET and EOG, that record eye movements), facial electromyography (EMG, for facial expressions), functional near-infrared spectroscopy (fNIRS, analysis of oxygenation level), or other physiological measures (e.g., heart rate, galvanic skin response).

**Figure 4:**
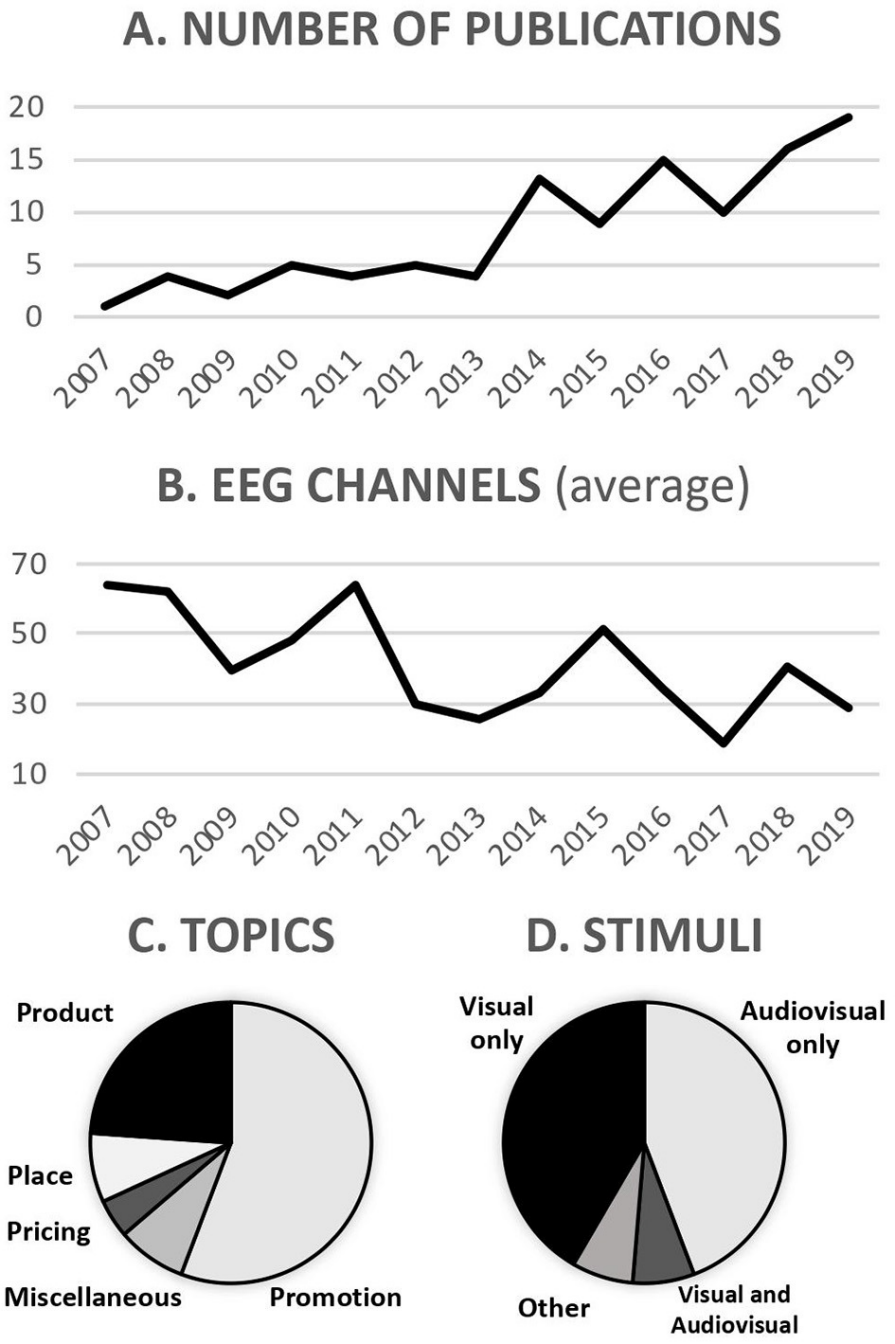
Literature review. **(A)** Number of publications between 2007 and 2019. **(B)** Average number of EEG channels between 2007 and 2019. **(C)** Distribution of research topics. **(D)** Distribution of stimuli used. Visual indicates static visual stimuli (e.g., picture), audiovisual indicates dynamic stimuli (e.g., commercial).

Some noteworthy trends (see Figure 4) include the increase in the usage of multiple concurrent techniques (from 6% of the papers in 2007 to 2011 to 36% in 2015 to 2019), and the reduction in the average number of electrodes used for the collection of EEG data (from 56 in 2006 to 2010 to 33 in 2015 to 2019), in addition to the steep increase in the overall number of publications (annual averages: 3 in 2006–2010, 14 in 2015–2019).

## Results

**Figure 5:**
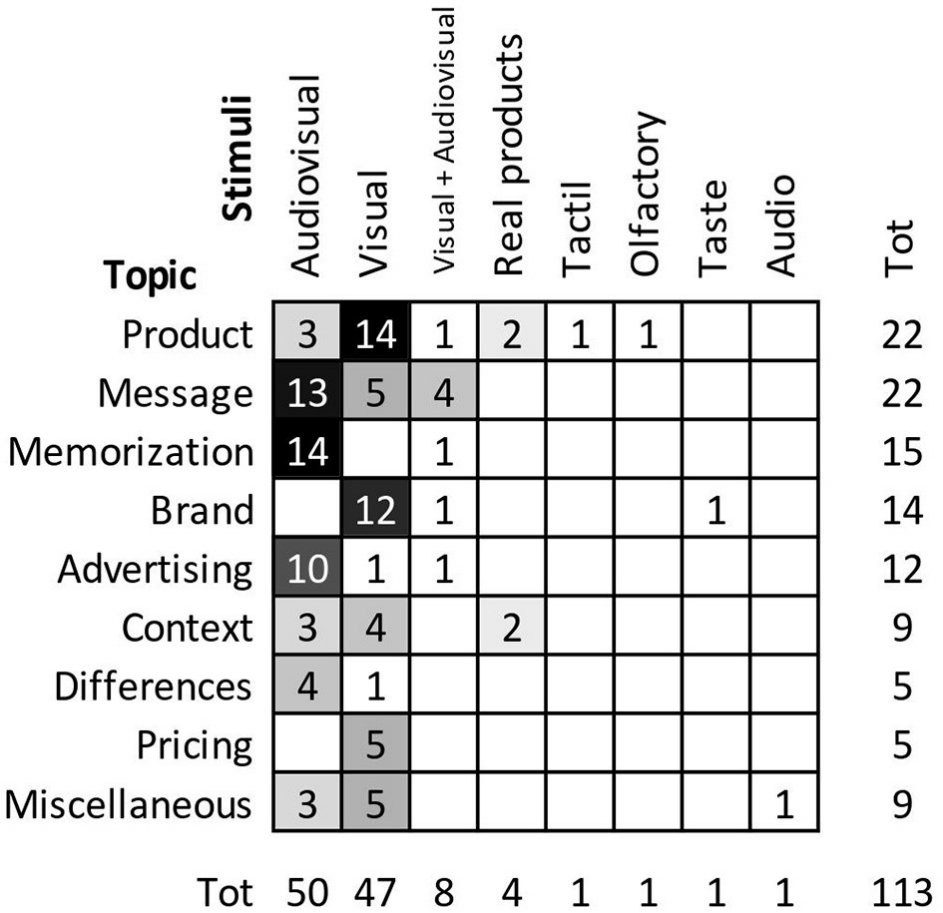
Distribution of articles based on research topic and type of stimuli used. Visual indicates static visual stimuli (e.g., picture), audiovisual indicates dynamic stimuli (e.g., commercial).

The four Ps of marketing offer a way to navigate the literature, also described in Table 1, by classifying the articles based on their main research question. This taxonomy is used for the rest of the section to introduce and discuss the main results in the existing literature. Figure 5 contains the distribution of articles in the review according to research topic and type of stimuli used (audio, visual, tactile, etc.). Visual and audiovisual stimuli have been widely used across topics, whereas others received less attention so far. We summarize the results in Table 3: for each category of stimulus, we include research and managerial implications, a reference paper, and we indicate the usual number of participants and EEG channels used.

**Table 3:** Research design, frameworks, and managerial implications.

**Stimuli Topic**	**Number of papers**	**Average # subjects**	**Average # EEG channels**	**Reference**	**Research Implications**	**Managerial Implications**
Visual	**47**	**27**	**32**	Gholami Doborjeh et al. (2018)	Multiple repetitions of visual stimuli (promotional messages, logos, price formats, etc.) represent a solid paradigm for EEG data collection. Event Related Potentials (ERP) analysis can be easily combined with pictures displayed multiple times. Additional techniques such as eye-tracking and galvanic skin conductance provide further insights and are frequently combined with EEG recording.	Static advertisements are commonly used in printed as well-online promotional messages. Similarly, the products displayed on a shelf in the supermarket are akin to the visual stimuli commonly used in experiments with EEG, often combined with behavioral measures, familiarity, and attention.Stimuli repetition paradigms can be helpful to test consumer loyalty.
Advertising	1	16	29	Guo et al. (2016)		
Brand	12	23	40	Yang (2018)		
Context	4	30	20	García-Madariaga et al. (2019)		
Differences	1	39	32	Jones et al. (2012)		
Message	5	28	25	Cuesta-cambra and Rodríguez-terceño (2017)		
Miscellaneous	5	27	32	Shen et al. (2018)		
Pricing	5	48	45	Ramsøy et al. (2018)		
Product	14	22	27	Khushaba et al. (2013)		
Audio-visual	**50**	**42**	**39**	Barnett and Cerf (2017)	Low-cost EEG equipment and support vector machine (SVM) techniques increase accuracy in terms of ranks and binary answers, reducing the sample size compared to traditional tools. Audiovisual stimuli can be used during natural memorizations tasks. EEG and additional techniques can investigate measurable physiological responses and reactions without relying on verbal reports.	EEG techniques are able to record brain activity with high temporal resolution and identify the reactions to TV commercials or other videos. Behavioral and neurophysiological measures allow to recognize the drivers of engagement and memorization, and select the parts of the commercials that are leading its success (or failure).Audiovisual stimuli are helpful for evaluating the novelty of a product/service, since they avoid the erosion of attention typical of stimuli repetition paradigms.
Advertising	10	33	39	Wei et al. (2018)		
Context	3	60	13	Rosenlacher et al. (2018)		
Differences	4	32	35	Cartocci et al. (2016)		
Memorization	14	47	53	Guixeres et al. (2017)		
Message	13	49	30	Harris et al. (2019)		
Miscellaneous	3	28	35	Boksem and Smidts (2015)		
Product	3	21	35	Dmochowski et al. (2014)		
Visual and audiovisual	**8**	**26**	**57**	Gordon et al. (2018)	EEG is a low invasive technique and can be combined with active and passive tasks from traditional marketing research, as well as novel tasks based on the replication of familiar environments and choice scenarios.	Behavioral and neurophysiological measures can be combined in the analysis of the effect on consumer behavior of price, promotions, displaying formats, and other variables of interest.
Advertising	1	30	14	Pileliene and Grigaliunaite (2017)		
Brand	1	26	32	Camarrone and Van Hulle (2019)		
Memorization	1	21	256	Daugherty et al. (2018)		
Message	4	25	36	Cartocci et al. (2017)		
Product	1	32	10	Ramsøy et al. (2019)		
Real products	**4**	**34**	**19**	Alvino et al. (2019)	EEG techniques can be used to measure the perception of products in multisensory settings and the impact of the surroundings on consumers' evaluation and choices. In this setting EEG can also be combined with additional techniques.	EEG measurements have been used to improve product display in the store. Despite the initial hope, EEG provides limited guidance in the phase of product development.
Context	**2**	**41**	**15**	Berčik et al. (2016)		
Product	**2**	**26**	**22**	Sargent et al. (2020)		
Audio miscellaneous	**1**	**26**	**32**	Lin et al. (2014)	Machine-learning methods improve the classification of emotions using EEG multimodal approach.	Voices, sounds and music play a key role in consumer engagement. Soundtracks, that are an essential part of TV and online ads, as well as jingles contribute to ads recall.
Tactile product	**1**	**24**	**64**	Hoefer et al. (2016)	Neurophysiologic measurements obtained with EEG may replace questionnaires in defining product perceived characteristics.	EEG can be implemented to test consumer pleasantness for all those products with a relevant tactile component (e.g., fabrics, mobile phones and covers, accessories, etc.).
Taste brand	**1**	**26**	**32**	Lucchiari and Pravettoni (2012)	Marketing actions such as brand manipulation can influence brain activity and change consumers' perception of a product. EEG provides objective measures of these effects.	Consumers often prefer branded products (including drugs) to the generic ones. EEG measures can be used to identify the role of a brand in developing consumption preferences and habits.

### Product

EEG techniques have been used in studies involving the evaluation of widely different products, and using one or multiple senses. The studies reviewed include wine tasting, scents smelling, textile touching, evaluation of food, clothes, and music, among the others. We consider separately the studies that explore perception and preferences over the characteristics of the products, and the studies that aim to identify the differences across market segments (e.g., divided by age, gender, or nationality).

#### Product Characteristics and Preferences

Marketers want to understand how the separate characteristics of a product contribute to the overall evaluation. In a study involving preferences over crackers, Khushaba et al. (2013) vary shape, flavor, and topping of the stimuli and ask the participants to indicate the preferred items. By combining behavioral, EEG, and eye-tracking data, the authors analyze the spectral activity associated with preferences (delta, alpha, and beta activity in the frontal area) and use mutual information analysis to identify the most salient attributes. Hoefer et al. (2016) worked in the same direction by using different fabrics and analyzing neurophysiological reactions toward tactile stimuli. By comparing vigilance (alpha-activity), emotional valence (alpha-asymmetry) and cognitive resources (ERP) across repeated trials, they can identify and interpret the consumers' preferences over fabrics.

#### Gender and Cultural Differences

Market segmentation is crucial for many firms, and marketers want to understand what drives differences between demographic groups. In a study involving TV advertisements and gender differences, Uva et al. (2015) adopt the LORETA analysis to identify differences in the brain activation between males and females while they watch video segments. Brain correlates are used to predict attention (P300 signal), cognitive processing (beta and gamma waves), and brain activity in different areas are associated with likeability and attractiveness (prefrontal cortex) and with emotion (prefrontal cortex and ACC). The systematic heterogeneity in the activations suggests structural differences in the way different market segments process ads (e.g., higher frequencies' power, ERPs' components, and anterior circulate cortex activation for female participants) and are used to assess the ad's effectiveness. In a similar study focused on cultural differences, Vecchiato et al. (2014a) show the Western and Asian versions of the same TV smartphone commercial to Italian and Chinese subjects. The two groups display no significant difference in attention or memorization (defined using theta and alpha signals), but pleasantness and emotion (theta and alpha signals, heart rate) depend on the cultural differences.

### Price

Neuroimaging techniques have been used to identify the adequate price for a product, especially when social or ethical aspects can be incorporated in the evaluation. In a study involving the pricing for various product categories (including apparel and fast-moving consumer goods) Ramsøy et al. (2018) find that willingness to pay correlates with prefrontal asymmetry in the gamma oscillation bands and with the trend in the beta frequency band during the first second of product viewing, a result that can be interpreted in terms of approach behavior and attentional gating. In a series of studies on corporate social responsibility and pricing, Lee (2016) displays different products accompanied by price and neutral or prosocial messages. The higher willingness to pay for the latter products can be explained by an empathic reaction to the stimulus. This result is supported by the correlation between product type and consumer empathy (theta-band activities of the anterior cingulate cortex).

### Promotion

The promotion of products and brands have been explored in many directions using visual and audiovisual stimuli in studies involving pleasantness and memorization of commercials, effectiveness of rational and emotional messages, and brand identity.

#### Advertising Rating and Pleasantness

After correctly designing the product and setting its price, it is crucial to promote it, for example through advertisement. One common experimental paradigm for EEG studies involves displaying a sequence of static images, several times each. In a study with frequently encountered objects, such as icons and logos, Handy et al. (2010) use ERPs to identify the emotional value. Stimuli are divided into liked and disliked ones, and the results show a significant effect of hedonic evaluation (central/parietal ERP component in the 150–200 ms post stimulus, and in the parietal/occipital ERP component in the 200–400 ms post stimulus) in a pattern that allows the classification of the pleasantness based on neurophysiological data. The paradigm can be adapted to analyze the characteristics of dynamic stimuli such as TV commercials, and use them as predictors of market-level response to advertising. Venkatraman et al. (2015) use a group of TV ads to compare the predictive power of different techniques (including EEG, eye tracking, and fMRI) and discuss how alpha activity (occipital alpha activity and frontal asymmetry) can be used to integrate the information from traditional self-reports to predict the aggregate real-world impact of the advertising campaign.

#### Advertising Attention and Memorization

The success of a commercial campaign depends also on whether the consumer will remember the promotional message after several days, and EEG patterns can be used as a measure of attention and predictor of memorization. In the first study on advertising memorization, Rossiter et al. (2001) investigate whether brain electrical can identify the video segments remembered 1 week later. One week after the first session (watch a documentary with short commercial interruptions) participants are shown various frames and are asked to indicate whether each frame was extracted from a commercial. Frames with the fastest and slowest SSVEP responses (steady-state visually evoked potential) are used for the memory test, and the scenes with fast SSVEP response in the left-hemisphere are more easily recognized. This paradigm has been extended to include repeated measures of the same commercial over a longer time span, with participants attending multiple sessions on five subsequent days, followed by an interview ten days later to determine which ads were remembered. Astolfi et al. (2008) adopt a head model and identify for the remembered ads significant differences in beta and gamma bands and higher spectral power in several cortical areas.

#### Rational and Emotional Messages

Advertisements usually contain both rational (such as product characteristics and benefits) and emotional parts (music, evocative scenes). Finding a good balance between them is a basic objective of effective communication. In order to understand the different brain activities associated with these two categories of messages, Cook et al. (2011) consider a series of ads with a clear prevalence of rational or emotional content. LORETA analysis shows different brain activation patterns according to the type of message. Rational messages are processed by the orbitofrontal cortex (involved in the evaluation of the rewards), whereas emotional messages activate the anterior cingulate cortex (attention and emotion processing), amygdala and hippocampus (vigilance and memory). In a study on the effectiveness of rational-based and action/emotion-based public health and social cause (HSC) advertisement, Harris et al. (2019) compare brain activity and survey data. They find theta synchronization and alpha desynchronization in the frontal area during all the campaigns, but alpha desynchronization appears in the parietal area only during one ad, suggesting that it would be the most effective one to adopt for the HSC campaign.

### Brand Identity

Promotion is not just advertising: companies invest resources to create and protect their own brand identity. Consumer neuroscience offers new tools to evaluate the potential of branding strategies and understand how brand familiarity generates value. For example, understand what are the mechanisms that make the consumers willing to choose the familiar brand instead of switching to a new private label. Brown et al. (2012) use a simple study with soft drinks to answer this question: even when the taste of a soft drink is identical, participants tend to prefer the known brand, reporting higher perceived pleasantness, showing low price elasticity, and displaying high amplitude value in the alpha spectrum over the frontal lobe area (associated with emotions). In a study on brand association, Camarrone and Van Hulle (2019) present a series of combinations of familiar brands and common words. The amplitude of the event-related potential components N400 indicates the incongruence between two concepts and is used to reveal associations between brands, product categories, and consumers' emotions. For example, the N400 response reveals a stronger association of the on-demand brand Netflix with relax-related words compared with TV-related ones.

### Place

A small number of articles studied the presentation of the product, instead of its attributes. In order to explore the influence of light and color on consumer behavior, Horská and Berčík (2014) introduced an experimental paradigm in a naturalistic environment. Participants wearing wireless EEG devices and cameras freely navigate a food store, while the products are presented under different lighting conditions. Light characteristics, including intensity and color temperature, can affect conscious and subconscious response to the visual stimuli (beta and alpha activity) and help the creation of positive emotional responses (measured by the asymmetry in the response between brain hemisphere), but the effect of light is different across demographic segments (the emotional response is mediated by age and gender). The social interaction with other consumers is another aspect of the purchase context, and Pozharliev et al. (2015) explore it in a highly innovative study on emotional experience with luxury products. The authors inquire whether consumers have a different response to luxury products when they are shopping with other people, compared with shopping alone. Pictures of various products (chocolate, beverages, shoes, lingerie) were classified into luxury or basic brands during a pre-test and used as stimuli for an observing task under two conditions. In the “alone” condition subjects participate individually, whereas in the “together” condition they are randomly paired with another participant. Luxury products score higher on the emotional value dimension (enhancement of the LPP amplitude) compared to basic brands, and ERP amplitudes associated with attention and motivational significance (P2 and P3 components in the visual cortex) occur more often in the together condition.

## Discussion

**Figure 6:**
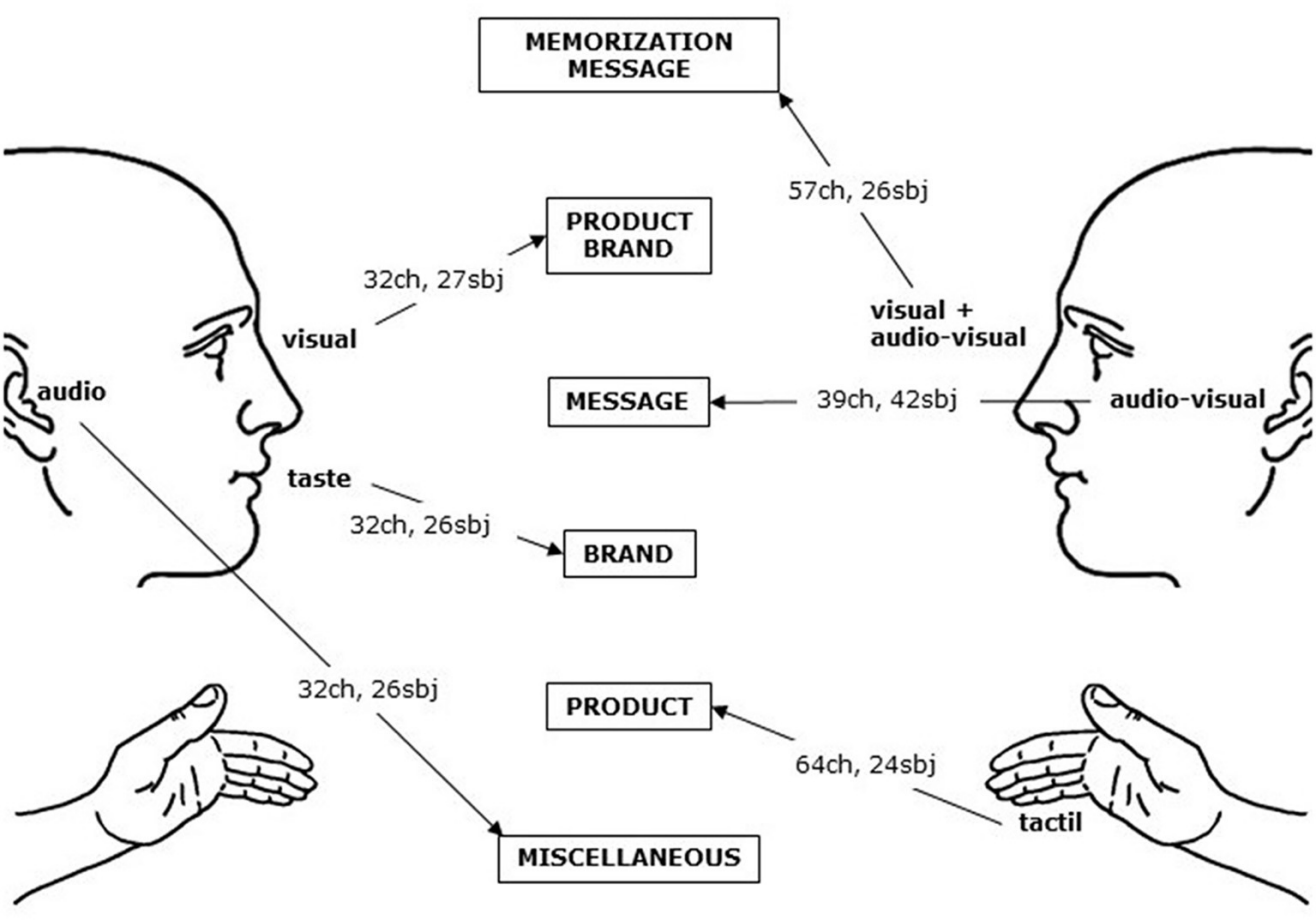
Average number of participants and EEG channels in the reviewed studies, grouped by stimulus category, and most frequent research topics for each category. Visual indicates static visual stimuli (e.g., picture), audiovisual indicates dynamic stimuli (e.g., commercial).

After summarizing the research and managerial implications emerging from the papers reviewed (Table 3), we provide now some guidelines to design EEG experiments (Figure 6) for both research and managerial applications. After emphasizing potentials and limitations of EEG in marketing research, we highlight some open questions in the literature that would benefit from EEG studies (Table 4).

**Table 4:** Research agenda.

**Topics**	**Key insights**	**Open questions**	**Added value brought by EEG**
Multisensory and immersive experiences	Promotional contents are expanding beyond traditional visual and audiovisual stimuli (TV ads, printed magazines, etc.), ranging from sophisticated home theater systems to 360 degrees “virtual reality” videos, and even including scents or tactile experiences, in order to offer the consumer multisensory and immersive experiences, which have been little studied so far.	Do immersive experiences surprise consumers because of their novelty only, or they provide a systematically more effective way to engage consumers? Can immersive experiences become a systematic way to engage consumers? Are multisensory stimuli more effective than the unisensory and traditional ones? Or do they end up overstimulating and confuse the consumers? Where is the balance?	EEG provides cardinal measures of engagement for both unisensory and multisensory stimuli. Pleasantness and surprise, among the main drivers of engagement, can be clearly individuated with EEG.
Consumer profiling	Behavioral differences in product rating and likelihood to purchase can be the result of multiple forces (willingness to pay, brand loyalty, curiosity, etc.) that are difficult to disentangle with traditional techniques.	How can we identify key differences across potential consumers? Do heterogeneous groups (based on personal data, socio-demographic and psychometric measures) display systematic differences in the way they understand, process, and react to promotional messages? Which message is more effective for different targets?	EEG data are not only complementary to questionnaires, but in an objective way they shed light on the neural processes underpinning consumers subjective answers.
Price formats	Pricing strategies include price level and format: gifts, add-ons, discounts, fixed or recurrent costs, are often used to differentiate products and encourage buyers to complete the purchase promptly.	Which price formats should be used to make a product or a service more appealing for new, recurrent, or loyal consumers? Do price formats affect the perception of price? Do they affect the process of search across alternative options? How do price formats affect the ease of comparison between similar products?	EEG provides objective measures of the effect of price on consumers, showing the neural correlates of rational and emotional reactions to price variations.
Social consumer neuroscience	Purchase decisions are rarely made in isolation. The same brain areas involved in self-referential cognition are indeed also those activated when buyers rely on other people's opinions and judgments, and interact with other customers, assistants, and experts. Mechanisms of word of mouth, trust, reputation, rating, and transmission of values play a major role in the purchase process (social facilitation). Little is known whether they are universal or domain-specific (e.g., necessities vs. luxury products).	How do recommendation modalities differ in their ability to convey information and convince consumers? Do they affect the purchase decision or (also) the future opinion about the product? Does the effectiveness of a suggestion depend on the product type, the person who gives the advice, or the consumers' current “state of mind?”	EEG can highlight the differences in brain activity during information processing according to the social communication modalities used.
Shopping in digital and physical stores	Digital and physical stores still coexist for many products, but it is unclear if there is complementarity or substitutability between them.	Do digital and physical stores cannibalize the same audience or strengthen the brand position? Should companies think of them as two different channels to reach the same buyers, or two different shopping experiences for different types of consumers? Which products should be sold (uniquely) in digital or physical stores?	EEG allows us to observe brain activity for different types of stimuli, including visual or audiovisual (digital stores), but also tactile and taste (physical stores).

*Overview of five research topics that would benefit from the adoption of neuroscientific techniques*.

*For each topic, the table contains a statement of its relevance in the current marketing literature, a list of open questions that traditional techniques may not be able to address, and an explanation of the value added to the research by collecting EEG data*.

### Exploring Non-conscious Behavior

Alongside with most neuroimaging approaches, EEG concedes the exploitation of brain processes bypassing the filter of conscious awareness. This notion becomes particularly relevant when dealing with decision-making processes in which non-conscious mechanisms are involved. As an example, using EEG and ERP, Camarrone and Van Hulle (2019) were able to reveal associations between brands, product categories, and emotions that would have otherwise gone unnoticed with conventional surveys. Further promising is the identification of EEG-derived indices as predictors of commercial success. Boksem and Smidts (2015) elegantly showed how individual self-declared willingness to pay is a poor predictor of population preference, while EEG gamma activity and medial-frontal beta power can well predict both individual preference and commercial success.

### Research Implications

The advantages of using EEG for marketing research can be summarized in four main points. First, the high temporal resolution of EEG is desirable for studies that involve videos (including TV commercials) and other dynamic stimuli (3D shapes and mobile apps), commonly adopted in conventional and unconventional marketing. The ability to investigate events spanning time windows on the order of milliseconds makes EEG particularly suitable for the investigation of relatively rapid neural and behavioral processes (Ratcliff et al., 2009). Second, EEG is convenient for naturalistic studies, as many EEG systems are portable and can be combined with other tools such as eye-tracking and wearable monitoring devices. In addition, increasingly used techniques such as hyperscanning turn a light on the promising fields of two-person neuroscience and social consumer neuroscience (Schilbach et al., 2013; Pozharliev et al., 2017). Third, EEG is minimally invasive, making recruitment of participants easier and their compliance higher, as compared to other neuroimaging tools (e.g., fMRI or MEG). On the one hand, this facilitates the involvement of large numbers of subjects, an essential advantage in the quest of robust results. On the other hand, the low invasiveness ensures that EEG protocols are more sustainable compared to other neuroscientific approaches, and therefore more likely to be approved by ethical committees. The resulting high compliance aids longitudinal studies based on repeated observations of the same pool of subjects to identify priming, habits, and learning effects. Fourth, EEG is considerably more affordable than other functional imaging approaches, like fMRI, and is accompanied by a long experimental literature (Brown and Behrmann, 2017).

### Managerial Implications

The technical and methodological advantages outlined above suggest how novel EEG results can aid managers in their strategic decisions. Companies are rarely willing to invest in a research project unless the results are expected to be clear and reliable. Neuroimaging research often does not lead to immediate indications about internal or external policy to implement (Ariely and Berns, 2010), but solid experimental designs might improve this. The difficulty in the interpretation of the results arises within the subject (as neural noise), but compared with traditional marketing tools these techniques allow to focus on general mechanisms underlying decision making, reducing other confounding factors such as market complexity (Karmarkar and Yoon, 2016). Since the very early days, the modulation of EEG power bands (the alpha one in particular) has been used as a proxy of attentional processes (Berger, 1929; Klimesch, 2012). A wide variety of well-investigated indices have since been developed and can offer an immediate application of EEG to the investigation of salient marketing stimuli. Another rather practical outcome relies on the objective comparison of the emotional charge of commercials. As in Ohme et al. (2010), EEG frontal asymmetries can be used as a diagnostic measure of emotional perception upon the exposure to different advertisements of the same product, assisting in the selection of the most appropriate stimuli. This helps marketers to go beyond verbal declarations of consumers. One more managerial implication of EEG application to marketing falls within the realm of pricing, as respondents tested using common self-reported verbal measures are more likely to give biased and socially acceptable answers (Nighswonger and Martin, 1981). Well-known research topics such as consumer profiling and price formats would benefit from the complementarity between new and old approaches. Several EEG measures including P300 ERPs and frontal asymmetries have been successfully applied in the identification of the willingness to pay in relation with consumers' expectations (Schaefer et al., 2016; Ramsøy et al., 2018). In parallel, EEG-based metrics, combined with behavioral observations, allow to recognize the drivers of engagement and memorization, familiarity, and loyalty, and therefore they can help in the selection of promising commercials. The adoption of multiple concurrent techniques, together with the knowledge accumulated through the last ten years of EEG experiments in marketing, is progressively leading to a less invasive approach. In recent years we have observed a reduction in the number of electrodes (Figure 4B) and an increase in the adoption of mobile devices.

### How to Design an EEG Study

We provide some basic guidelines that should help those readers who are approaching this kind of study for the first time to orient themselves in experimental design. We are well aware that the complexity of designing an experiment with EEG is far from being easily subsumed into fragmented wisdom pills. However, these general indications should provide some initial resources for a non-expert reader. Starting with your research question, you should first choose the type of stimulus. Figure 6 displays the average number of participants and EEG electrodes used in the studies included in this review, grouped by type of stimulus (see also Figure 5 and Table 3). These numbers should not be interpreted as optimal for every study, and you should select the number of participants based on a power estimation analysis. In addition, reference papers reported in Table 2 and in Table 3 (e.g., Ramsøy et al., 2019) contain several examples of well-designed studies. The current literature provides little information about the use of multisensorial or interactive stimuli. We expect to see in the future more research activities exploring these aspects (see Areas for future research), and advise to consult methodological papers (e.g., Cline et al., 2018) to address the challenge of setting new standards. After the data collection, EEG data require a pre-processing phase, which is rather homogeneous across the studies we reviewed, as far as the adoption of analysis toolboxes (Delorme and Makeig, 2004). Finally, the choice of a suitable analysis technique almost entirely depends on the type of information the researcher aims to derive from the available data. This decision usually requires solid expertise.

### Limitations

The results of the papers reviewed do not always provide conclusive evidence, or they emphasize the contingent results in a way that is difficult to generalize. Results from studies that focus on methodological aspects are often difficult to apply to practical scenarios. These issues lead us to strongly advise merging different competences in the research group that is designing the experiments and analyzing the results. Collaborative efforts can indeed balance the correct approach to consumer neuroscience, made of rigorous methodology applied to questions relevant to marketing. Companies are already able to interpret the results of conventional marketing studies and decide whether they are aligned with the expectations. Instead, brain imaging results are harder to explain and to apply to the crucial variables for companies. As a result, many firms are skeptical about consumer neuroscience research and not all promises have been fulfilled. The authors believe that it is possible to overcome skepticism by pointing out that brain imaging can go beyond the boundaries of any behavioral study and offer insights into the mechanisms of unconscious decision-making process, but certain conditions must be met in order to make it worthwhile. Designing experiments of consumer neuroscience according to protocols of common practice in scientific literature (e.g., randomized control trail, RCT), researchers and marketers who apply consumer neuroscience techniques might provide additional reasons to companies to overcome their skepticism. Indeed, the definition of precise research questions and of standardized protocols for treatments, control groups or stimuli, and for robustness checks might improve the reliability and replicability of the studies in this emerging field and make findings clearer also to non-experts.

### Areas for Future Research

In this review we have highlighted the extensive use of consumer neuroscience techniques in studies with visual and audiovisual stimuli. The high time resolution of EEG markers and the adoption of objective indices for decision-making processes can be extended further, to more complex multisensory stimuli and immersive experiences, including virtual reality environments. Plausible prospects regarding future consumer neuroscience research include an increased spread of research questions toward the consumer's interaction with the environment, including physical characteristics of the store, and social interactions (experts, friends, or other consumers, e.g., Pozharliev et al., 2017). The few recent articles that explore these areas successfully combine well-designed experiments and clear managerial implications. Research on pricing should move away from a “willingness to pay” approach, that can be successfully explored with traditional techniques, and focus on market segmentations and drivers of the perceived value. This novel approach seems promising in the studies that use environmental-friendly products and communication strategies based on corporate social responsibility as stimuli and suggests that a broader scope is possible. Finally, user experience in digital and physical environments—and the difference between the two—deserves broader space for future consumer neuroscience research. The opportunity to investigate the subconscious processes underlying the purchase behavior seems a natural complement to the high amount of data about online search and purchase. For example, to the best of our knowledge, there is no study exploring the search process within and between online shopping platforms.

Neuro-psycho-physiological, biometric, psychometric, and behavioral data can be jointly used to move beyond the limits of subjective and self-declared tools, such as questionnaires. However, brain imaging techniques (and other combined tools) are not intended to replace traditional surveys. On the one hand, we underlined the advantages of exploring hybrid solutions between neuroscientific and more consolidated marketing tools. On the other hand, the current lack of conclusive findings on the most promising EEG metrics, or the rationale of their combination, for the prediction of decision-making, might be attributable to an inappropriate experimental design rather than to the limits of the technique itself. For this reason, we provided basic suggestions to design an experiment with EEG. For instance, ERPs need several repetitions of the stimulus to be registered, thus compromising the novelty of a stimulus. The use of a neutral video interspersed with a repeated stimulus (e.g., a picture) over time can prevent this undesired effect. We advise against extremely specific paradigms whose results are difficult to be generalized and exported to adjacent scenarios, as well as against too-broad research questions that do not help researchers, and even companies, in a concrete way. Table 4 offers a list of five areas for future research, which would benefit from an interdisciplinary approach. We also give a brief explanation of how EEG would provide a valid technique to explore these plausible perspectives. The research areas satisfy three criteria: (1) neuroscientific techniques can provide new evidence with respect to traditional ones; (2) the current literature provides little or no answers to them; and (3) they also address direct managerial implications.

## Conclusion

This review has shown the limits of the current literature: we do not advise blind optimism, and we claim that successful research and trust from companies will depend on achieving the suggested improvements. Traditional marketing techniques capture customers' conscious actions and choices, whereas neuroimaging tries to offer objective and replicable measures of neural processes occurring during decision-making. The relatively low cost, minimally-invasiveness, and portability are the key points that make EEG a suitable tool for both lab and field experiments, providing results and recommendations that can improve marketing actions. The long path of EEG since 1929 and the extensive use of this technique in the field of neuroscience might offer a wide range of markers of attention, cognition, and emotions to be applied to naturalistic stimuli such as advertisements. We believe that a complete and rigorous consumer neuroscience project requires joint contributions from an interdisciplinary group. From a methodological point of view, we expect research projects to be designed like clinical studies: sample selection criteria, information allowing replicability, selection of additional techniques, and data acquisition, filtering, preprocessing and cleaning, need to be finely tuned to each study and well-reported in the final report. Research questions should be precisely defined and include treatment, control, and robustness checks according to the protocol of a randomized control trial (RCT), as common practice in experimental scientific literature.

## Data Availability Statement

The raw data supporting the conclusions of this article will be made available by the authors, without undue reservation.

## Author Contributions

AB, SR, UF, and GT contributed to conceive and design this systematic review. SR, AB, and LT conducted the study selection, they extracted the data from the selected articles, and discussed among them in case of controversy on the classification of articles. SR ran the data analysis. AB and SR drafted the manuscript with the supervision from UF and GT. AB conceived the conceptual framework and under the supervision of UF edited the technical part of the manuscript related to EEG technique. All authors contributed to the interpretation of data and to the final version of this article, that they all hence approved to be submitted.

## Conflict of Interest

The authors declare that the research was conducted in the absence of any commercial or financial relationships that could be construed as a potential conflict of interest.
